# Worldwide Prevalence of Human Papillomavirus and Relative Risk of Prostate Cancer: A Meta-analysis

**DOI:** 10.1038/srep14667

**Published:** 2015-10-06

**Authors:** Lin Yang, Shuanghua Xie, Xiaoshuang Feng, Yuheng Chen, Tongzhang Zheng, Min Dai, Cindy Ke Zhou, Zhibin Hu, Ni Li, Dong Hang

**Affiliations:** 1 National Office for Cancer Prevention and Control, Cancer Institute and Hospital, Chinese Academy of Medical Sciences/Peking Union Medical College, Beijing, China; 2 Department of hospital infection control, Beijing Jishuitan Hospital, Fourth Medical College of Peking University, Beijing, China; 3 School of Public Health, Brown University, Providence, RI, USA; 4 Division of Cancer Epidemiology and Genetics, National Cancer Institute, Maryland, USA; 5 Department of Epidemiology and Biostatistics, School of Public Health, Nanjing Medical University, China.

## Abstract

Despite the increasing number of studies conducted recently to evaluate the association between HPV infections and the risk of prostate cancer, the results remain inconclusive. Furthermore, the prevalence and distribution of overall and individual HPV types worldwide in prostate cancer has not been reported until now. Therefore, we estimated the prevalence of HPV in prostate cancer by pooling data of 46 studies with 4919 prostate cancer cases, taking into account the heterogeneity of major related parameters, including study region, specimen type, HPV DNA source, detection method, publication calendar period and Gleason score. Moreover, we tested the association of HPV infections with prostate cancer risks by a meta-analysis of 26 tissue-based case-control studies. We found that the prevalence of HPV infection was 18.93% (95% CI = 17.84–20.05%) in prostate cancer cases, and most of which were high-risk HPV types (17.73%, 95% CI = 16.52–18.99%). The prevalence varied by region, PCR primers used, publication calendar period and Gleason score. Our study also showed a significantly increased risk of prostate cancer with the positivity of overall HPV detected in prostate tissues (OR = 1.79, 95% CI = 1.29–2.49) and revealed the geographic variation of association strength (*P* < 0.001). In conclusion, HPV infections may contribute to the risk of prostate cancer.

Human papillomavirus (HPV) is most commonly transmitted through sexual activity. Carcinogenetic types of HPV, such as HPV 16 and 18, have been proved to be a necessary cause of invasive cervical cancer^1^. Studies have also suggested possible links between HPV infections and other female cancers, such as vulva^2^, vagina^2^ and breast^3^. In addition to cancers specific to females, HPV has also been shown to be associated with the risk of cancers in male anogenital and urinary sites, such as penis^2^, anus^2^ and bladder^4^.

Studies have found positive associations of prostate cancer with sexual activities and sexually transmitted diseases^5^. It has been hypothesized that the prostate gland can also be infected by HPV for its anatomic proximity of the anogenital and urinary sites. In 1992, Rabkin *et al.* reported men with anal cancer, a disease that has been associated with HPV, had an increased risk for developing subsequent prostate cancer^6^. Then, a series of epidemiological and laboratory studies detected HPV in malignant, benign or normal prostate tissues. However, results on the association between HPV infections and prostate cancer risks remain controversial.

We retrieved published data on HPV prevalence and combined individual studies on the association between HPV infections and prostate cancer risks through a meta-analysis. We further evaluated how influential parameters (such as study region, sample source, detection method and publication calendar period) affected the results from the meta-analysis.

## Results

Overall, 46^5^,^7^,^8^,^9^,^10^,^11^,^12^,^13^,^14^,^15^,^16^,^17^,^18^,^19^,^20^,^21^,^22^,^23^,^24^,^25^,^26^,^27^,^28^,^29^,^30^,^31^,^32^,^33^,^34^,^35^,^36^,^37^,^38^,^39^,^40^,^41^,^42^,^43^,^44^,^45^,^46^,^47^,^48^,^49^,^50^,^51^ publications were included in the present study, among which 32^8^,^9^,^10^,^11^,^12^,^15^,^16^,^17^,^20^,^22^,^23^,^24^,^25^,^26^,^27^,^28^,^29^,^30^,^31^,^33^,^34^,^35^,^36^,^37^,^38^,^44^,^46^,^47^,^48^,^49^,^50^,^51^ and 11^5^,^7^,^14^,^18^,^19^,^21^,^32^,^39^,^41^,^42^,^43^ respectively reported HPV prevalence in tissues and sera of prostate cancer cases, as well as 3^13^,^40^,^45^ presented data on both tissues and sera. In addition, one study^40^ was conducted in both African-American and Italian men, and therefore, data in this study were consequently divided into two subgroups when stratified analyses were done by study region. Therefore, 25 countries and/or regions presented data on HPV prevalence in 6365 prostate cancer cases (Supplementary Table 1). Since 5 studies^5^,^7^,^13^,^14^,^31^ only presented data of individual HPV type, a total of 4919 and 5547 (Table 1) men from the 6365 prostate cancer cases were used to estimate the prevalence of overall HPV and HPV 16, respectively.

**Figure 1:**
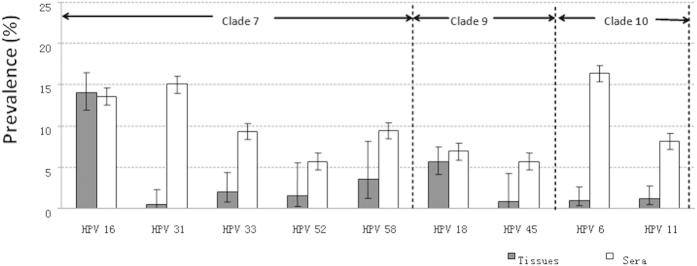
Prevalence of Individual HPV Types in Tissues and Sera.

The prevalence of HPV ranged from 0% to 76.92% (Supplementary Table 1) and yielded an average of 18.93% (95% CI = 17.84–20.05%) (Table 1) and 17.18% (95% CI = 15.66–18.81%) after adjusted for study region, specimen type and publication calendar period. Thirteen HPV types (HPV6, 11, 16, 18, 31, 33, 35, 39, 45, 52, 58, 59 and 68) were detected in prostate cancer cases across studies. The prevalence of high-risk HPV types (17.73%, 95% CI = 16.52–18.99%) was much higher than that of low-risk HPV types (4.31%, 95% CI = 2.84–6.25%) (OR = 4.78, 95% CI = 3.20–7.45) (Table 1). Furthermore, the prevalence of HPV clade A9 (17.20%, 95% CI = 16.08–18.37%) was higher than that of HPV clade A7 (6.60%, 95% CI = 5.92–7.34%) and clade A10 (3.44%, 95% CI = 2.17–5.17%) (Table 1) (OR_A9/A7_ = 2.94, 95% CI = 2.55–3.38 and OR _A9/A10_ = 5.83, 95% CI = 3.78–9.43, respectively). The 5 most common high-risk HPV types identified, in order of decreasing prevalence, were HPV16 (13.68%, 95% CI = 12.89–14.62%), HPV31 (11.82%, 95% CI = 9.73–14.17%), HPV33 (8.39%, 95% CI = 7.26–9.63%), HPV18 (6.60%, 95% CI = 5.92–7.34%) and HPV58 (3.55%, 95% CI = 1.16–8.08%). HPV11 and HPV6 were also common in prostate cancer with the prevalence of 2.34% (95% CI = 1.29–3.90%) and 1.02% (95% CI = 0.28–2.60%) (Table 1). Further analysis showed the prevalence of all the individual HPV types based on sera was consistently higher than that on tissues but HPV 16. HPV 16 was the most common type in tissues but ranked the third in sera. Meanwhile, HPV 18 was the second common type when detected in tissues but ranked the sixth in sera (Fig. 1).

After stratified by study region, we found that overall HPV prevalence in prostate cancer cases was highest in Africa (68.29%, 95% CI = 61.45–74.60%) with only one study, followed by Asia (20.25%, 95% CI = 16.74–24.13%) and Latin America (18.63%, 95% CI = 12.94–25.52%) (Table 1). The HPV prevalence in prostate cancer cases was significantly higher (OR = 1.29, 95% CI = 1.03–1.63) from studies published in 2000–2015 (19.43%, 95% CI = 18.25–20.65%) than those in 1990–1999 (15.74%, 95% CI = 13.06–18.73%) (Table 1). In addition, the cancer cases with high Gleason score (≥7) had higher (OR = 1.40, 95% CI = 1.12–1.75) HPV prevalence (18.70%, 95% CI  = 16.28–21.32%) than those with low Gleason score (<7, 14.10%, 95% CI = 12.42–15.91%) (Table 1).

For studies with HPV DNA detection in prostate cancer tissues, there was no significant difference of HPV prevalence between using fresh (17.13%, 95% CI = 14.04–20.59%) and fixed tissues (16.71%, 95% CI = 14.18–19.47%) (OR = 1.03, 95% CI = 0.76–1.39) (Table 2). With respect to HPV detection methods in prostate cancer tissues, the broad spectrum PCR primers, type–specific PCR primers, and both combined, as well as non–PCR methods, namely *in situ* hybridization (ISH), immunohistochemistry and HC2, were used. HPV prevalence was 17.72% (95% CI = 15.84–19.72%) when PCR-based method was used but only 5.36% (95% CI = 1.12–14.87%) when non-PCR-based method was used. Further analysis showed that the type-specific PCR primers were generally demonstrated to be more efficient with a detection rate of 27.34% (95% CI = 22.28–32.86%) (Table 2). Meanwhile, the HPV prevalence was again significantly higher (OR = 1.70, 95% CI = 1.12–2.58) in Gleason score ≥7 (22.68%, 95% CI = 18.16–27.73%) than those in Gleason score <7 (14.73%, 95% CI = 11.20–18.86%), when HPV DNA was detected in tissues (Table 1).

Thirty eight case-control studies^8^,^9^,^10^,^12^,^13^,^16^,^17^,^18^,^19^,^20^,^21^,^22^,^23^,^24^,^25^,^26^,^27^,^28^,^29^,^30^,^32^,^35^,^36^,^37^,^38^,^39^,^40^,^41^,^42^,^43^,^44^,^45^,^46^,^47^,^48^,^49^,^50^,^51^ with 4741 prostate cancer cases and 7074 controls were retrieved to estimate overall HPV and prostate cancer risks, among which 8^18^,^19^,^21^,^32^,^39^,^41^,^42^,^43^ and 28^8^,^9^,^10^,^12^,^13^,^16^,^17^,^20^,^22^,^23^,^24^,^25^,^26^,^27^,^28^,^29^,^30^,^35^,^36^,^37^,^38^,^44^,^46^,^47^,^48^,^49^,^50^,^51^ case-control studies respectively used HPV serology and DNA detection in prostate tissues, as well as 2^40^,^45^ studies used both of them. We additionally excluded 4^16^,^25^,^40^,^50^ studies because of the absence of HPV in both cases and controls. This resulted in 34 case-control^8^,^9^,^10^,^12^,^13^,^17^,^18^,^19^,^20^,^21^,^22^,^23^,^24^,^26^,^27^,^28^,^29^,^30^,^32^,^35^,^36^,^37^,^38^,^39^,^41^,^42^,^43^,^44^,^45^,^46^,^47^,^48^,^49^,^51^ studies with 4540 prostate cancer cases and 6892 controls in our analysis for the association between overall HPV infections and prostate cancer risks, among which 8^18^,^19^,^21^,^32^,^39^,^41^,^42^,^43^ and 25^8^,^9^,^10^,^12^,^13^,^17^,^20^,^22^,^23^,^24^,^26^,^27^,^28^,^29^,^30^,^35^,^36^,^37^,^38^,^44^,^49^,^51^ case-control studies respectively used HPV serology and DNA detection in prostate tissues, as well as 1 study^45^ used both of them (Table 3).

**Figure 2:**
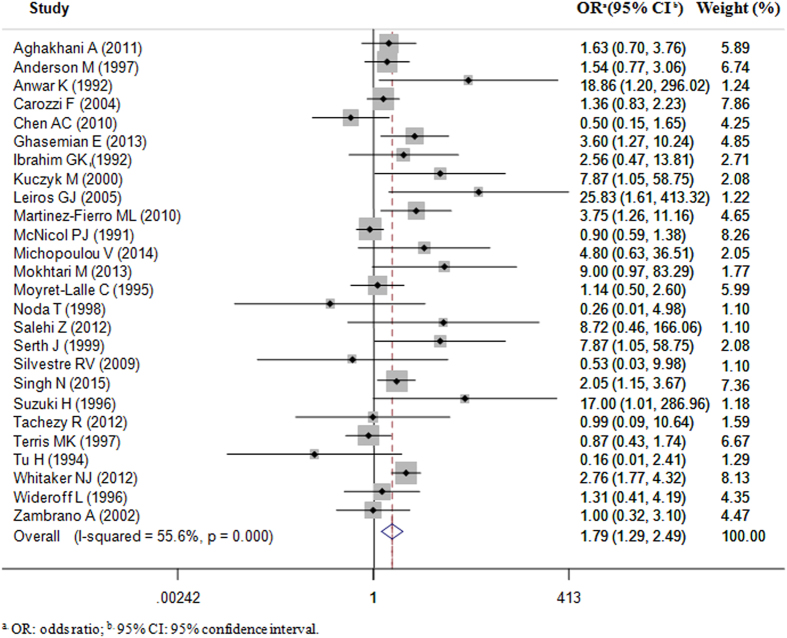
Odds Ratios of Prostate Cancer Cases Associated with HPV in Tissues (Cases vs. Controls), by Random-effect Model.

Overall, infection of any type of HPV was significantly associated with an increased risk of prostate cancer (OR = 1.32, 95% CI = 1.12–1.55) with significant between-studies heterogeneity (*Q* = 73.87, *P*<0.001). However, the statistically significant association was absent for studies based on sera (OR = 1.03, 95% CI = 0.95–1.12) while persisted in studies based on tissues (OR = 1.79, 95% CI = 1.29–2.49). Hence, the following analyses focused on 26 studies^8^,^9^,^10^,^12^,^13^,^17^,^20^,^22^,^23^,^24^,^26^,^27^,^28^,^29^,^30^,^35^,^36^,^37^,^38^,^44^,^49^,^51^ with 1218 prostate cancer cases and 1346 controls with HPV DNA detection in tissues. According to the result of the heterogeneity test (*Q* = 56.29, *P* < 0.001), the random-effect model was chosen to evaluate the pooled OR (Fig. 2 and Table 3). Generally, there was a significantly increased prostate cancer risk in relation to infection of any type of HPV (OR = 1.79, 95% CI = 1.29–2.49) (Fig. 2 and Table 3). Egger’s and Begg’s tests, which were used to indicate publication bias, proved to be insignificant (*P* = 0.133 and 0.261, respectively). In subgroup analysis, we found the geographic heterogeneity of the magnitude of association estimates between overall HPV infections and prostate cancer risks (*P* for between-strata heterogeneity < 0.001) and revealed that the increased risk of prostate cancer with HPV infections was evident in Europe (OR = 1.95, 95% CI = 1.35–2.81), Asia (OR = 2.82, 95% CI = 1.89–4.21) and Latin America (OR = 5.76, 95% CI = 2.25–14.76). However, there was no significant heterogeneity for OR estimates across the other subgroups.

## Discussion

Epidemiological and biological studies have now conclusively proved that a variety of infectious agents are the major causes of cancers worldwide^52^. In the last two decades, at least six different viruses have been linked to the development of specific types of human cancers. HPV, one of the most important infectious agents, has been shown to be linked to penile cancer^2^, arousing research interests in male genital and urinary systems. The prostate inflammation resulting from sexually transmitted infections in the course of carcinogenesis has been speculated^53^.

Three meta-analyses have been published on the correlation between HPV infections and the risk of prostate cancer^54^,^55^,^56^, but the results were not consistent. The first analysis in 2005^55^ with 10 studies found a significantly increased risk of prostate cancer in relation to HPV infections (OR = 1.39, 95% CI = 1.12–2.06) by combining studies in tissues and sera together. The latter two studies in 2011^54^ and 2015^56^, which mainly focused on the infection of the most common oncogenic types (HPV 16 and/or HPV 18) in relation to prostate cancer, found that the overall risk of prostate cancer was not significantly increased by either HPV 16 (OR = 1.09, 95% CI = 0.97–1.23) or HPV 18 (OR = 1.05, 95% CI = 0.89–1.24) infection when HPV was detected in sera and tissues combined^54^, but significantly increased when HPV DNA detected in prostate tissues^54^,^56^.

Whether HPV infections are associated with increased risk of prostate cancer, and if so, whether the bio-samples used for detection would affect the results still need to be answered. In addition, although positive associations have been reported in previous meta-analyses, no study, until today, has comprehensively accessed the type-specific prevalence of HPV infections in prostate cancer by region, detection method and publication year, which might provide supportive information for the wider use of HPV vaccine in addition to its use in cervical cancer prevention. Hence, in our present study, with the updated data and more detailed analyses, we analyzed the relationship between HPV infections and the risk of prostate cancer, particularly by detection method, and provided evidence for the link between HPV infections and increased risk of prostate cancer. The link was prominent in studies with HPV detected in tissues. Furthermore, we revealed the geographic variations in the association strengths and emphasized other methodological parameters (e.g., detection method) in further analyses that have never been shown in the previous studies.

In general, the prevalence of overall and individual HPV types was higher when detected in sera than in tissues except HPV 16. The distribution of individual types also varied despite bio-samples used in HPV detection (Fig. 1). This suggested that the HPV prevalence in sera was not well corresponding to that in tissues. The higher prevalence in sera might be due to HPV infections from anatomical sites other than prostate. The results also suggested the caveat of sero-epidemiological studies on HPV detection, such as the indistinguishable origins of the antibody from other mucosal sites of the body and the complex link between seropositivity and historical infections and current disease status. Hence, further analyses in prostate cancer tissues merit more attention.

Further analysis focused on cancer tissues showed that PCR-based method with the type-specific primer was more suitable in HPV DNA detection in prostate cancer cases. Comparing with the low detection rate of HPV DNA when PCR amplification of long DNA fragments in the *L1* gene is adopted, HPV type-specific primers are usually designed to amplify shorter sequences of HPV DNA and might be more sensitive to detect HPV DNA sequences^4^. Therefore, it is possible that the detection rate for HPV using HPV type-specific PCR primers may be higher than other PCR methods. On the other hand, type-specific PCR based method for HPV detection in prostate tissues might be more useful, partly due to the low copy numbers of HPV DNA in prostate cancer tissues.

Our study also suggested a moderate geographical variation in HPV prevalence and association strengths with prostate cancer. Meta-analyses on the prevalence of cervical HPV DNA worldwide showed a higher HPV detection rate in Africa, and a lower prevalence in North America and Europe^57^. So higher HPV prevalence in prostate cancers in Africa (68.29%, 95% CI = 61.45–74.60%) is expected. Meanwhile, the risk of prostate cancer by HPV infection was highest in Latin America (OR = 5.76, 95% CI = 2.25–14.76) and Asia (OR = 2.82, 95% CI = 1.89–4.21) (no cases-control comparison for mem with Africa origin). Since Asia has the lowest prostate cancer incidence and mortality, this may due to the moderate risk magnitude and the complex risk profiles (e.g., hygienic habits, sexual and smoking behaviors) for prostate cancer.

The prevalence of HPV was higher (OR = 1.29, 95% CI = 1.03–1.63) in studies published in 2000–2015 (19.43%, 95% CI = 18.25–20.65%) than those in 1990–1999 (15.74%, 95% CI = 13.06–18.73%). The increased prevalence is expected and it is primarily due to improved HPV detection protocols. Since the majority of histological type of prostate cancer is adenocarcinoma, it has been hypothesized that HPV 18 to be more predominant than HPV 16 as that in cervical adenocarcinoma^58^, which was similar to the HPV type distribution in breast carcinoma^4^. However, the present study showed that HPV 16 was the most common type among all types included. It also should be noted that the prevalence of clade 10 (3.94%, 95% CI = 1.82–7.36%) was lower either than HPV 6 (16.35%, 95% CI = 9.82–24.88%) or HPV 11 (8.13%, 95% CI = 5.43–11.61%) in sera. The reason was that the data on prevalence of clade 10 could not be extracted from 2 publications^13^,^59^, which only presented data of individual HPV types in case of the occurrence of multiple infections. However, these 2 studies^13^,^59^ presented relatively higher prevalence of HPV 6 (24.1% and 18.9%, respectively) and 11 (12.5% and 18.9%, respectively) in sera. Thus, the prevalence of HPV clade 10 in sera was under-estimated in the present study.

In addition to the prostate, HPV are also discussed in the pathogenesis of anogenital and urinary cancers, including penis, anus, bladder, testis and renal cancer. HPV appears not to play a major causative role in renal^60^,^61^ and testicular carcinogenesis^62^ for the failure detection of HPV DNA in cancer cases. On the contrary, there is sufficient evidence in humans for the carcinogenicity of certain HPV types, (i.e. HPV 16 and 18) associated anal and penile cancers^2^. About 90% of anal squamous cell cancers occur in individuals with detectable HPV infections^63^. Of those, HPV 16 and/or HPV 18 are detectable in more than 90% of cases^63^. Although the etiology of penile cancer is still unclear, approximately 40% of all penile tumors are thought to be attributable to HPV infections^2^. A quantitative review of studies that used PCR methods for HPV DNA detection found HPV presented in 45.4% of invasive penile tumors after adjusting for PCR primer, histology subtype, and year and geographical location of the study^64^. However, HPV infections should be kept in mind regarding cases of bladder cancer, and prostate cancer. The prevalence of HPV in prostate cancer cases (17.18%) was lower than that in the anal and penile cancers, while it is similar to the HPV prevalence (16.88%) in bladder cancer cases (most occurred in males) reported in our previous meta-analysis^4^.

Very limited studies exploring the association between HPV infections and main clinical features of prostate cancer, e.g., cancer types and prostate-specific antigen (PSA) levels, made it difficult to do further sub-analysis by these features in the present meta-analysis. Most of the cancer cases were adenocarcinoma. Only one study^14^ showed that sero-positivity of HPV 18 was associated with 92% increased risk of adenocarcinoma (OR = 2.92, 95% CI = 1.15–7.38), and the association was slightly attenuated (OR = 2.59, 95% CI = 1.17–5.75) when all histologic tumor types were included (127 adenocarcinoma, 14 unspecified carcinoma and 1 transitiocellular carcinoma). Since 2 case-control studies^24^,^38^ reported the null association of HPV infections to PSA levels, the prevalence of HPV might be irrelevant to PSA levels based on the present evidence. However, this present meta-analysis indicated a higher prevalence of HPV infections in high-grade prostate cancer (Gleason score ≥7). Due to the retrospective temporality in case-control studies, whether HPV infections precede prostate cancer carcinogenesis or tumor environment is amiable for HPV invasion needs to be confirmed in prospective studies. Moreover, no study has explored the association between HPV infections and hormone response in prostate cancer till today.

The mechanism of HPV infections and prostate cancer development is far from clear. It has been proposed that exposure to environmental factors such as infectious agents and dietary carcinogens, and hormonal imbalances lead to injury of the prostate gland and to the development of chronic inflammation and regenerative ‘risk factor’ lesions, referred to as proliferative inflammatory atrophy (PIA)^65^.Two meta-analyses with the statistically significant evidence of the association between prostatitis and prostate cancer^66^,^67^ also suggest that inflammation resulting from infections may be one mechanism for prostate cancer carcinogenesis. Although a case-control study has showed that HPV infections are not related with prostatitis-related symptoms when urethral swab was used for HPV detection^68^, another study has showed that HPV was identified in the prostatic secretions from patients with type III prostatitis and might be associated with the degree of intraprostatic inflammation^69^. Whether HPV infections associated with the risk of prostate cancer is mediated by chronic prostatic inflammation which leads to initiation and progression of prostate cancer, needs further functional research.

Until today, prostate cancer is not clearly linked to any preventable risk factors^70^. Although the causal involvement of HPV in prostate carcinogenesis is still a matter of controversial debate, the association of HPV infections with prostate cancer, if substantiated, would be unexpectedly good news for cancer prevention^14^.

## Conclusions

Generally, the present study suggested the link between HPV infections and prostate cancer, though the risk estimates varied by study region. Furthermore, as we are aware, it is the first study to summarize the HPV prevalence and the distribution of individual oncogenic types worldwide. Our study highlighted the importance of detection methods in studies of HPV infections and prostate cancer. Considering the great variation in HPV prevalence and the risk estimates by the influential parameters, multi-center large-scale prospective studies are needed. In addition, the etiological and biology functional researches on HPV infections and prostate cancer are necessary.

## Materials and Methods

### Study selection

We used Medline to search for relevant articles published from January 1989 to May 2015 using the MeSH terms “Papillomavirus”, “Human”, and “Prostate cancer”. We also evaluated citations in retrieved articles. The work flow is shown in the Supplementary Figure 1. We tried to include all studies on HPV DNA or antibodies detected in biopsy tissues or sera. We included the most recent study when multiple reports were published with substantial overlaps^26^,^45^. The exclusion criteria were as follows: (i) studies on immunosuppression patients, for example, patients after renal and cardiac transplantation; (ii) case reports; (iii) publications not in English; (iv) studies without extractable data from the original article.

### Data extraction

Two reviewers (Lin Yang and Shuanghua Xie) independently extracted data from selected articles according to a standard form created *a priori* for this study. Disagreement was resolved by consensus. For each study included, the following information was extracted: first author, year of publication, country of origin, specimens type (tissue or serum), HPV DNA source (fixed or fresh), detection method (PCR or not, and types of primers), sample size, HPV prevalence overall by disease status (case or control) and type-specific: HPV 6, 11, 16, 18, 31, 33, 35, 39, 45, 52, 58, 59 and 68), and matching criteria if controls were present. When HPV was assessed in both prostate tissues and sera, only the results obtained from tissues was used^13^,^40^,^45^. Detailed information on all included studies was presented in supplementary Table 1.

Gleason score of prostate cancer cases was also extracted. The majority of studies classified prostate cancers into two subgroups as high (≥7) and low (<7) Gleason scores. However, 2 studies^32^,^34^ categorized Gleason score into 2–4, 5–7 and 8–10. We collapsed the two lower categories in to the group of low Gleason score in the present meta-analysis. Additionally, data of HPV infections and Gleason score in one study^29^ was excluded because the classification of Gleason score was not compatible with the other studies and the data could not be merged.

### Statistical Analyses

We described characteristics of included studies, and calculated prevalence and its 95% confidence intervals of overall, clade-specific (clade A7: HPV 18 and 39; clade A9: HPV 16, 31, 33, 35, 52, 58; clade A10: HPV 6 and 11) and type–specific HPV prevalence (11 high-risk HPV types: HPV 16, 18, 31, 33, 35, 39, 45, 52, 58, 59 and 68; 2 low-risk HPV types: HPV 6 and 11) in total prostate cancers and by detection method. An unconditional logistic regression model was used to adjust and compare the HPV prevalence by the influential parameters including study region, specimen type and published year.

We also pooled risk estimates for HPV infections and prostate cancer risk using included case-control studies. A fix-effect or random-effect model was used to pool the data, based on the Mantel-Haenszel method and the DerSimonian and Laird method, respectively. These two models provide similar results when between-studies heterogeneity is absent; otherwise, random-effect model is more appropriate. Between-studies heterogeneity test was performed by using the *χ*^2^-based *Q* test, and the heterogeneity was considered significant if *P* < 0.05. Publication bias was evaluated using Egger’s linear regression asymmetry test^71^ and Begg’s rank correlation test^72^. All analyses were performed using the Stata statistical software (version 11.0, StataCorp, College Station, TX).

## Additional Information

**How to cite this article**: Yang, L. *et al.* Worldwide Prevalence of Human Papillomavirus and Relative Risk of Prostate Cancer: A Meta-analysis. *Sci. Rep.* **5**, 14667; doi: 10.1038/srep14667 (2015).

## Supplementary Material

Supplementary Information

## Figures and Tables

**Table 1:** HPV Prevalence in Prostate Cancer Cases Based on Tissues and Sera, across Region, Publication Calendar Period, Gleason Score and HPV Type.

	**Total**			**Tissue–based detection**			**Serum–based detection**			***P***
	**No. of studies**	**No. of cases**	**HPV Prevalence (%) (95% CI**^a^)	**No. of studies**	**No. of cases**	**HPV Prevalence (%) (95% CI)**	**No. of studies**	**No. of cases**	**HPV Prevalence (%) (95% CI)**	
**Total**	46	4919	18.93 (17.84–20.05)	35	1597	17.28 (15.46–19.23)	14	3436	19.70 (18.38–21.07)	**0.041**
**Region**										
North America	17	2702	16.69 (15.30–18.15)	10	384	11.20 (8.22–14.79)	7	2318	17.60 (16.07–19.21)	**0.002**
Europe	14	1146	15.18 (13.15–17.39)	11	347	19.30 (15.29–23.86)	4	850	15.06 (12.72–17.64)	0.071
Asia	9	479	20.25 (16.74–24.13)	9	479	20.25 (16.74–24.13)	0	0	NA	NA
Africa	1	205	68.29 (61.45–74.60)	0	0	NA	1	205	68.29 (61.45–74.60)	NA
Latin America	3	161	18.63 (12.94–25.52)	3	161	18.63 (12.94–25.52)	0	0	NA	NA
Ocean	3	226	17.26 (12.57–22.83)	3	226	17.26 (12.57–22.83)	1	53	NA	NA
Mixed	0	NA^b^	NA	0	0	NA	1	63	1.59 (0.04–8.53)	NA
**Publication calendar period**										
1990–1999	18	667	15.74 (13.06–18.73)	16	620	16.45 (13.62–19.61)	3	110	3.64 (1.00–9.05)	**<0.001**
2000–2015	28	4252	19.43 (18.25–20.65)	19	977	17.81 (15.46–20.36)	11	3326	20.23 (18.88–21.64)	0.094
**Gleason score**										
**<7**	16	1582	14.10 (12.42–15.91)	13	353	14.73 (11.20-18.86)	3	1229	13.91 (12.03-15.98)	0.697
**≥7**	16	957	18.70 (16.28–21.32)	13	313	22.68 (18.16-27.73)	3	644	16.77 (13.96-19.88)	0.028
**HPV Type**										
High-risk^c^	32	3745	17.73 (16.52–18.99)	25	1039	18.19 (15.89–20.67)	9	2820	17.38 (15.99–18.83)	0.556
Low-risk^d^	11	603	4.31 (2.84–6.25)	10	438	3.88 (2.28–6.14)	3	279	7.17 (4.43–10.85)	0.052
**Individual Type**										
Clade 9	32	4273	17.20 (16.08–18.37)	23	987	14.89 (12.73–17.27)	10	3349	17.59 (16.31–18.92)	**0.048**
HPV16	33	5547	13.68 (12.89–14.62)	22	954	14.05 (11.90–16.41)	14	4760	13.57 (12.61–14.57)	0.697
HPV31	7	855	11.82 (9.73–14.17)	6	239	0.42 (0.01–2.31)	3	720	15.00 (12.47–17.82)	**<0.001**
HPV33	12	2194	8.39 (7.26–9.63)	8	301	1.99 (0.73–4.29)	6	1997	9.31 (8.07–10.67)	**<0.001**
HPV35	5	189	0.53 (0.01–2.91)	5	189	0.53 (0.01–2.91)	0	0	NA	NA
HPV52	3	129	1.55 (0.19–5.49)	3	129	1.55 (0.19–5.49)	1	53	5.66 (1.18–15.66)	0.123
HPV58	4	141	3.55 (1.16–8.08)	4	141	3.55 (1.16–8.08)	1	53	9.43(3.13–20.66)	0.098
Clade 7	26	4848	6.60 (5.92–7.34)	19	829	5.67 (4.20–7.47)	7	4019	6.79 (6.03–7.61)	0.236
HPV18	25	4815	6.60 (5.92–7.34)	18	796	5.65 (4.15–7.49)	9	4123	6.89 (6.13–7.70)	0.202
HPV39	2	105	0.00 (0.00–3.45)	2	105	0.00 (0.00–3.45)	0	0	NA	NA
HPV45	3	131	0.76 (0.02–4.18)	3	131	0.76 (0.02–4.18)	1	53	5.66 (1.18–15.66)	**0.039**
HPV59	1	65	0.00 (0.00–5.52)	1	65	0.00 (0.00–5.52)	0	0	NA	NA
HPV68	1	65	0.00 (0.00–5.52)	1	65	0.00 (0.00–5.52)	0	0	NA	NA
Clade 10	11	639	3.44 (2.17–5.17)	10	474	2.74 (1.47–4.64)	2	228	3.94 (1.82–7.36)	0.391
HPV6	7	391	1.02 (0.28–2.60)	7	391	1.02 (0.28–2.60)	2	104	16.35 (9.82–24.88)	**<0.001**
HPV11	9	598	2.34 (1.29–3.90)	8	433	1.15 (0.38–2.67)	4	332	8.13 (5.43–11.61)	**<0.001**

^a^95% CI: 95% confidence interval.

^b^NA: Not available.

^c^High-risk: HPV16,18,31,33,35,39,45,52,58,59 and 68.

^d^Low-risk: HPV6 and 11.

**Table 2:** HPV Prevalence in Prostate Cancer Cases by DNA Source and PCR Primers used for HPV DND Detection.

	**No. of Studies**	**No. of Cases**	**HPV Prevalence (%) (95% CI**^**a**^)	**OR(95% CI**^a^)
HPV DNA specimen				
Fixed tissue	17	802	16.71 (14.18–19.47)	1.00
Fresh tissue	16	537	17.13 (14.04–20.59)	1.03 (0.76–1.39)
Mixed or unclear	4	258	19.38 (14.74–24.74)	1.20 (0.82–1.74)
Detection method				
PCR-based	32	1541	17.72 (15.84–19.72)	
Broad spectrum primers	19	967	12.62 (10.59–14.88)	1.00
Type-specific primers	8	289	27.34 (22.28–32.86)	**2.61 (1.86–3.63)**
Combination of broad spectrum and type-specific primers	5	285	25.26 (20.32–30.73)	**2.34 (1.66–3.29)**
Non-PCR-based^b^	3	56	5.36 (1.12–14.87)	

^a^95% CI: 95% Confidence interval.

^b^Non-PCR-based: *In situ* hybridization, Immunohistochemistry and HC2.

**Table 3:** Meta-analysis of Case-control Studies on the Risk of Prostate Cancer Tissues and Sera, Stratified by Region, Publication Calendar Period and HPV Type.

	**Total**				**Tissue-based detection**				**Serum-based detection**			
	**No. of studies**	**No. of cases**	***P***** for heterogeneity**	**OR** a **(95% CI** b)	**No. of studies**	**No. of cases**	***P***** for heterogeneity**	**OR (95% CI)**	**No. of studies**	**No. of cases**	***P***** for heterogeneity**	**OR (95% CI)**
Total	34	4540	<0.001	**1.32 (1.12–1.55)**	26	1218	<0.001	**1.79 (1.29–2.49)**	10	3436	0.302	1.03 (0.95–1.12)
Region												
North America	12	2543	0.883	1.01 (0.90–1.14)	6	225	0.632	0.98 (0.70–1.36)	6	2318	0.813	1.02 (0.90–1.15)
Europe	8	1057	0.075	1.11 (0.93–1.32)	7	258	0.180	**1.95 (1.35–2.81)**	2	850	0.665	0.94 (0.79–1.13)
Asia	8	473	0.162	**2.82 (1.89–4.21)**	8	473	0.162	**2.82 (1.89–4.21)**	0	0	NA	NA
Africa	1	205	NA^c^	NA	0	0	NA	NA	1	205	NA	NA
Latin America	3	161	0.119	**5.76 (2.25–14.76)**	3	161	0.119	**5.76 (2.25–14.76)**	0	0	NA	NA
Ocean	2	101	0.008	1.29 (0.25–6.80)	2	101	0.008	1.29 (0.25–6.80)	0	0	NA	NA
Mixed	0	0	NA	NA	0	0	NA	NA	1	63	NA	NA
Publication calendar period												
1990–1999	12	494	0.029	1.37 (0.85–2.21)	11	447	0.018	1.38 (0.82–2.30)	2	110	0.257	0.71 (0.20–2.50)
2000–2015	22	4046	<0.001	**1.34 (1.12–1.59)**	15	771	0.036	**2.13 (1.45–3.12)**	8	3326	0.243	1.04 (0.96–1.12)
HPV Type												
High-risk^d^	24	3525	0.006	**1.19 (1.00–1.42)**	17	819	<0.001	**1.70 (1.03–2.82)**	9	2820	0.383	1.06 (0.96–1.16)
Low-risk^e^	5	331	0.534	0.75 (0.38–1.47)	5	331	0.534	0.75 (0.38–1.47)	1	51	NA	NA
Related to HPV16												
Yes	24	4090	0.018	1.15 (0.96–1.36)	16	768	0.001	**1.95 (1.06–3.57)**	10	3436	0.424	1.05(0.96–1.16)
No	12	3157	0.283	1.10 (0.94–1.29)	8	409	0.066	**1.75 (1.22–2.51)**	5	2799	0.962	0.98 (0.84–1.16)
Related to HPV18												
Yes	12	3181	0.284	1.02 (0.82–1.28)	8	433	0.094	1.82 (0.97–3.41)	5	2799	0.606	0.92 (0.73–1.17)
No	16	3318	0.149	1.08 (0.96–1.21)	12	570	0.054	**1.60 (1.23–2.09)**	5	2799	0.895	0.98 (0.87–1.11)

^a^OR: Odds ratio.

^b^95% CI: 95% confidence interval.

^c^NA: Not available.

^d^High-risk: HPV 16, 18, 31, 33, 35, 39, 45, 52, 58, 59 and 68.

^e^Low-risk: HPV 6 and 11.
