# Supplementary material for: Worldwide Prevalence of Human Papillomavirus and Relative Risk of Prostate Cancer: A Meta-analysis
Source: Sci Rep. 2015 Oct 6;5:14667. doi: 10.1038/srep14667 (PMC4594101; doi:10.1038/srep14667)
Supplement: Supplementary Information [file srep14667-s1.doc]

**Worldwide Prevalence of Human Papillomavirus and Relative Risk of Prostate Cancer: A Meta-analysis**

Lin Yang^1,2^, Shuanghua Xie^1^, Xiaoshuang Feng^1^, Yuheng Chen^1^, Tongzhang Zheng^3^, Min Dai^1^, Cindy Ke Zhou^4^, Zhibin Hu^5^, Dong Hang^5*^, Ni LI^1*^

1. National Office for Cancer Prevention and Control, Cancer Institute and Hospital, Chinese Academy of Medical Sciences, Peking Union Medical College, Beijing, China
2. Department of hospital infection control, Beijing Jishuitan Hospital, Fourth Medical College of Peking University, Beijing, China
3. School of Public Health, Brown University, Providence, RI, USA

4. Division of Cancer Epidemiology and Genetics, National Cancer Institute,

Maryland, USA

5. Department of Epidemiology and Biostatistics, School of Public Health, Nanjing Medical University, China

Lin Yang and Shuanghua Xie contributed equally to this work.

**Correspondence to:** Dr. Ni Li, National Office for Cancer Prevention and Control, Cancer Institute and Hospital, Chinese Academy of Medical Sciences, No. 17 Panjiayuannanli, Chaoyang District, Beijing, 100021 P.R. China.

Tel: +86-10-8778-8662. Fax: +86-10-8778-7394. Email: [lini1240@hotmail.com](mailto:lini1240@hotmail.com);

and Dr. Dong Hang, Department of Epidemiology and Biostatistics, School of Public Health, Nanjing Medical University, 140 Hanzhong Road, Nanjing 210029, China. Tel (Fax): 86-25-8686-8438. Fax: +86-25-8686-8439. Email: handkuku@163.com

| **Supplementary table 1.**  **Studies Included in the Meta-analysis and Their Characteristics by Region** | | | | | | | | | | | | | | | | | | | | | | | | | | | | | | | | | | | | | | | | | | | | | |  |
| --- | --- | --- | --- | --- | --- | --- | --- | --- | --- | --- | --- | --- | --- | --- | --- | --- | --- | --- | --- | --- | --- | --- | --- | --- | --- | --- | --- | --- | --- | --- | --- | --- | --- | --- | --- | --- | --- | --- | --- | --- | --- | --- | --- | --- | --- | --- |
| **First Author** | **Year of publication** | | | **Region** | | **Sample Source** | | **Detection Method** | | **HPV Types Detected** | **Match** | | **No. of Case**  **(% of HPV+)** | **No. of control(% of HPV+)** | | **Prevalence of HPV types detected in prostate cancer** | | | | | | | | | | | | | | | | | | | | | | | | | | | | | |  |
|  |  |  |  |  |  |  |  |  |  |  |  |  |  |  |  | **HPV**  **6**  **(%)** | | **HPV 11**  **(%)** | | **HPV 16**  **(%)** | **HPV 18**  **(%)** | | **HPV 31**  **(%)** | | | **HPV 33**  **(%)** | | | **HPV 35**  **(%)** | | | **HPV 39**  **(%)** | | **HPV 45**  **(%)** | | | **HPV 52**  **(%)** | **HPV 58**  **(%)** | | **HPV 59**  **(%)** | | | | | **HPV 68**  **(%)** |  |
| ***Asia*** | | | | | | | | | | | | | | | | | | | | | | | | | | | | | | | | | | | | | | | | | | | | | |  |
| Aghakhani A | | 2011 | | Iran | | Biopsy, fixed tissue | | PCR | | 6,11,16,18 | BPH | | 104  （12.5） | 104  （7.69） | | 0.96 | | 1.92 | 6.73 | | | 2.88 | |  | | |  | | |  | | |  | |  |  | | |  | |  | | |  | | |
| Anwar K | | 1992 | | Japan | | Biopsy, fixed tissue | | PCR | | 16,18,33 | BPH | | 58  (44.83） | 20  （0.00） | |  | |  | 17.24 | | | 27.59 | |  | | | 8.62 | | |  | | |  | |  |  | | |  | |  | | |  | | |
| Gazzaz FS | | 2009 | | Saudi | | Biopsy, fresh tissue | | HC2 | | 18 types of HPV | BPH | | 6  (0.00) | 50  (0.00) | | 0 | | 0 | 0 | | | 0 | | 0 | | | 0 | | | 0 | | | 0 | | 0 | 0 | | | 0 | | 0 | | | 0 | | |
| Ghasemian E, | | | 2013 | Iran | Biopsy, fixed tissue | | PCR | |  | | BPH | | 29  (17.20) | 167  (4.79) | | |  |  |  | | |  | |  | | |  | | |  | | |  | |  |  | | |  | |  | | |  | | |
| [Mokhtari](http://www.ncbi.nlm.nih.gov/pubmed/?term=Mokhtari M%5Bauth%5D) M | | | 2013 | Iran | Biopsy, fixed tissue | | Immunohistochemical | |  | | BPH | | 30  (10.00) | 90  (1.10) | | |  |  |  | | |  | |  | | |  | | |  | | |  | |  |  | | |  | |  | | |  | | |
| Noda T | | | 1998 | Japan | Biopsy, fixed tissue | | PCR | | 16,18,31,33,35,52,58 | | BPH | | 38  （0.00） | 71  （4.23） | | |  |  | 0 | | | 0 | | 0 | | | 0 | | | 0 | | |  | |  | 0 | | | 0 | |  | | |  | | |
| Salehi Z | | | 2012 | Iran | Biopsy, fresh tissue | | PCR | |  | | BPH | | 68  (4.41) | 85  (0.00) | | |  |  |  | | |  | |  | | |  | | |  | | |  | |  |  | | |  | |  | | |  | | |
| Singh N | | | 2015 | Indian |  | | PCR | | 6,11,16,18 | | BPH | | 95  (41.05) | 55  (20.00) | | | 2.11 | 1.05 | 31.58 | | | 6.32 | |  | | |  | | |  | | |  | |  |  | | |  | |  | | |  | | |
| Suzuki H | | | 1996 | Japan | Biopsy, fresh tissue | | PCR | | 16 | | normal prostate and 22 metastatic cancer tissues | | 51  （15.69） | 51  （0.00） | | |  |  | 15.69 | | |  | |  | | |  | | |  | | |  | |  |  | | |  | |  | | |  | | |
| ***Europe*** | | | | | | | | | | | | | | | | | | | | | | | | | | | | | | | | | | | | | | | | | | | | | |  |
| Adami HO | | 2003 | | Sweden | | Blood | | ELISA | | 16,18,33 |  | | 238 ^a^ | 210 ^a^ |  | | |  | 13.03 | | | 11.76 | |  | | | 29.41 | | |  | | |  | |  |  | | |  | |  | | |  | | |
| Anderson M | | 1997 | | United Kingdom | | Biopsy, fresh tissue | | PCR | | 16 | BPH | | 13（76.92） | 10  (50.00） |  | | |  | 0 | | |  | |  | | |  | | |  | | |  | |  |  | | |  | |  | | |  | | |
| Balis V | | 2007 | | Greece | | Biopsy, 22fixed;20fresh | | PCR | | 11,16,18,33 | |  | 42  （4.76） |  |  | | | 0 | 0 | | | 0 | |  | | | 0 | | |  | | |  | |  |  | | |  | |  | | |  | | |
| Carozzi F | | 2004 | | Italy | | Biopsy, fixed tissue | | PCR | | 6,11,16,18,31,33,35,45,52,58 | BPH | | 26（65.38） | 25  (48.00） | 3.85 | | | 0 | 11.54 | | | 11.54 | | 0 | | | 0 | | | 3.85 | | |  | | 3.85 | 7.69 | | | 15.38 | |  | | |  | | |
| Dillner J | | 1998 | | Finland | | Blood | | ELISA | | 11,16,18,33 | |  | 165 ^a^ | 290 ^a^ |  | | | 5.45 | 4.24 | | | 10.3 | |  | | | 4.24 | | |  | | |  | |  |  | | |  | |  | | |  | | |
| Korodi Z | | 2005 | | Nordic | | Blood | | ELISA | | 16,18,33 |  | | 799  (13.39) | 2596  (13.98) |  | | |  | 5.88 | | | 2.88 | |  | | | 6.38 | | |  | | |  | |  |  | | |  | |  | | |  | | |
| Kuczyk M | | 2000 | | Germany | | Biopsy, fresh tissue | | PCR | | 16 | BPH | | 47  (21.28) | 37  (2.70) |  | | |  | 21.28 | | |  | |  | | |  | | |  | | |  | |  |  | | |  | |  | | |  | | |
| Michopoulou V | 2014 | | | Greece | | Biopsy, fixed tissue | | PCR | | 37 high- and low-risk | Non-prostate cancer | | 50  (16.00) | 30  (3.40) |  | | |  | 4.00 | | | 8.00 | | 2.00 | | |  | | |  | | |  | |  |  | | |  | |  | | |  | | |
| Moyret-Lalle C | 1995 | | | France | | Biopsy, fresh tissue | | PCR | | 16,18 | Adenoma hyperplasias | | 27  (33.33) | 24  (29.17) |  | | |  | 33.33 | | | 0 | |  | | |  | | |  | | |  | |  |  | | |  | |  | | |  | | |
| Rotola A | | 1992 | | Italy | | Biopsy, fresh tissue | | PCR | | 6/11,16 | Non-neoplastic tissues | | 8 ^a^ | 17 ^a^ |  | | |  | 75 | | |  | |  | | |  | | |  | | |  | |  |  | | |  | |  | | |  | | |
| Rogler A | | 2011 | | Germany | | Biopsy, fixed tissue | | PCR | |  |  | | 33  (0.00) |  |  | | |  |  | | |  | |  | | |  | | |  | | |  | |  |  | | |  | |  | | |  | | |
| Serth J | | 1999 | | Germany | | Biopsy, fresh tissue | | PCR | | 16 | BPH | | 47  (21.28) | 37  (2.70) |  | | |  | 21.28 | | |  | |  | | |  | | |  | | |  | |  |  | | |  | |  | | |  | | |
| Strickler HD | | 1998 | | Italy | | Biopsy, fresh tissue | | PCR | |  | BPH | | 14  (0.00) | 13  (0.00) |  | | |  |  | | |  | |  | | |  | | |  | | |  | |  |  | | |  | |  | | |  | | |
|  | |  | |  | |  | |  | |  |  | |  |  |  | | |  |  | | |  | |  | | |  | | |  | | |  | |  |  | | |  | |  | | |  | | |
| Tachezy R | | 2012 | | czech | | Biopsy, fixed tissue | | PCR | |  | BPH | | 48  (2.08) | 95  (2.11) |  | | |  |  | | |  | |  | | |  | | |  | | |  | |  |  | | |  | |  | | |  | | |
|  | |  | |  | | Blood | | ELISA | | 6,11,16,18,31,33 | BPH | | 51  (41.18) | 172  (47.09) | 13.73 | | | 15.69 | 7.84 | | | 5.88 | | 7.84 | | | 11.76 | | |  | | |  | |  |  | | |  | |  | | |  | | |
| ***Oceania*** | | | | | | | | | | | | | | | | | | | | | | | | | | | | | | | | | | | | | | | | | | | | | |  |
| Chen AC | | 2010 | | Australia | | Blood | | fluorescent | | multiple |  | | 53 ^a^ | 104 ^a^ | 18.87 | | | 18.87 | 9.43 | | | 5.66 | | | 7.55 | | | 3.77 | | |  | |  | | 5.66 | 5.66 | | | 9.43 | |  | | |  | | |
|  | |  | |  | | Biopsy, fresh tissue | | PCR | | 18 | BPH | | 51  (13.73) | 11  (27.27) |  | | |  |  | | | 13.73 | | |  | | |  | | |  | |  | |  |  | | |  | |  | | |  | | |
| Melissa A Yow, | | 2014 | | Australia | | Biopsy, fixed tissue | | PCR | |  | Adjacent normal tissue | | 115  (0.00) | 51  (0.00) |  | | |  |  | | |  | | |  | | |  | | |  | |  | |  |  | | |  | |  | | |  | | |
| Whitaker NJ | | 2012 | | Australia | | Biopsy, fixed tissue | | PCR | |  | BPH + normal tissue | | 50  (58.00) | 100  (21.00) |  | | |  |  | | |  | | |  | | |  | | |  | |  | |  |  | | |  | |  | | |  | | |
|  | |  | |  | | Biopsy, fresh tissue | | PCR | | 18 |  | | 10  (33.33) |  |  | | |  |  | | | 10  (33.33) | | |  | | |  | | |  | |  | |  |  | | |  | |  | | |  | | |
| ***Africa*** | | | | | | | | | | | | | | | | | | | | | | | | | | | | | | | | | | | | | | | | | | | | | |  |
| Sitas F | | 2007 | | South African | | Blood | | ELISA | | 16 |  | | 205  (68.29) | 673  (57.5) |  | | |  | 68.29 | | |  | |  | | |  | | |  | | |  | |  |  | | |  | |  | |  | | | |
| ***North America*** | | | | | | | | | | | | | | | | | | | | | | | | | | | | | | | | | | | | | | | | | | | | | |  |
| Effert PJ | | 1992 | | US | | Biopsy, fixed tissue | | PCR | | 16,18 |  | | 30  (0.00) |  |  | | |  | 0 | | | 0 | |  | | |  | | |  | | |  | |  |  | | |  | |  | |  | | | |
| Hayes RB | | 2000 | | US | | Blood | | ELISA | | 16 |  | | 274  (9.49) | 289  (8.65) |  | | |  | 9.49 | | |  | |  | | |  | | |  | | |  | |  |  | | |  | |  | |  | | | |
| Hisada M | | 2000 | | US | | Blood | | ELISA | | 16 |  | | 48  (41.67) | 63  (30.16) |  | | |  | 41.67 | | |  | |  | | |  | | |  | | |  | |  |  | | |  | |  | |  | | | |
| Huang WY | | 2008 | | US | | Blood | | ELISA | | 16,18 |  | | 868 ^a^ | 1281 ^a^ |  | | |  | 14.63 | | | 13.25 | |  | | |  | | |  | | |  | |  |  | | |  | |  | |  | | | |
| Ibrahim GK | | 1992 | | US | | Biopsy, 23fresh | | PCR | | 6,11,16,18,31,33,35,39,45 | |  | 23  (13.04) |  | 0 | | | 0 | 13.04 | | | 0 | | 0 | | | 0 | | | 0 | | | 0 | | 0 |  | | |  | |  | |  | | | |
|  | |  | |  | | Biopsy, 46fixed | | PCR | | 6,11,16,18,31,33,35,39,45 | BPH | | 17  (17.65) | 29  (6.90) | 0 | | | 0 | 17.65 | | | 0 | | 0 | | | 0 | | |  | | |  | |  |  | | |  | |  | |  | | | |
| Masood S | | 1991 | | US | | Biopsy, fixed tissue | | in situ hybridization | | 6,11,16,18,31,33,35 | Glandular hyperplasia | | 20  (0.00) | 20  (0.00) | 0 | | | 0 | 0 | | | 0 | | 0 | | | 0 | | |  | | |  | |  |  | | |  | |  | |  | | | |
| McNicol PJ | | 1991 | | Canada | | Biopsy, fresh tissue | | PCR | | 16,18 | BPH + normal tissue | | 27  (51.85) | 61  (57.38) |  | | |  | 51.85 | | | 3.7 | |  | | |  | | |  | | |  | |  |  | | |  | |  | |  | | | |
| Rosenblatt KA | 2003 | | | US | | Blood | | ELISAs | | 16,18 |  | | 642  (11.21) | 570  (10.53) |  | | |  | 9.19 | | | 3.43 | |  | | |  | | |  | | |  | |  |  | | |  | |  | |  | | | |
| Saad F | | 1999 | | Canada | | Biopsy, fresh tissue | | PCR | |  |  | | 40  (0.00) |  |  | | |  |  | | |  | |  | | |  | | |  | | |  | |  |  | | |  | |  | |  | | | |
| Strickler HD | | 1998 | | US | | Blood | | ELISAs | | 16 | Endocrine disorders | | 47  (6.38) | 48  (4.17) |  | | |  | 6.38 | | |  | |  | | |  | | |  | | |  | |  |  | | |  | |  | |  | | | |
| Strickler HD | | 1998 | | African-American | | Biopsy, fresh tissue | | PCR | |  | BPH | | 46  (0.00) | 48  (0.00) |  | | |  |  | | |  | |  | | |  | | |  | | |  | |  |  | | |  | |  | |  | | | |
| Sutcliffe S | | 2010 | | US | | Blood | | ELISAs | | 16,18,31 | Non-prostate cancer | | 616  (29.22) | 616  (29.22) |  | | |  | 18.34 | | | 4.22 | | 16.23 | | |  | | |  | | |  | |  |  | | |  | |  | |  | | | |
| Sutcliffe S | | 2007 | | US | | Blood | | ELISAs | | 16,18,33 | Non-prostate cancer, | | 691  (15.48) | 691  (16.5) |  | | |  | 7.53 | | | 6.08 | |  | | | 7.24 | | |  | | |  | |  |  | | |  | |  | |  | | | |
| Terris MK | | 1997 | | US | | Biopsy, fixed tissue | | PCR | | 16 | BPH | | 53  (18.87) | 78  (21.79) |  | | |  | 18.87 | | |  | |  | | |  | | |  | | |  | |  |  | | |  | |  | |  | | | |
| Tu H | | 1994 | | US | | Biopsy, fixed tissue | | PCR | | 16,18 |  | | 60  (3.33) | 1  (0.00) |  | | |  | 1.67 | | | 1.67 | |  | | |  | | |  | | |  | |  |  | | |  | |  | |  | | | |
| Wideroff L | | 1996 | | US | |  | | PCR | | 6,11,16,18,31,33,45; | hyperplastic | | 56  (12.50) | 42  (9.52) |  | | |  |  | | |  | |  | | |  | | |  | | |  | |  |  | | |  | |  | |  | | | |
| Zambrano A | | 2002 | | US | | Biopsy, fresh tissue | | PCR | | 16,18,33,58 | Non-cancer | | 12  (33.33) | 12  (33.33) |  | | |  | 8.33 | | | 8.33 | |  | | | 8.33 | | |  | | |  | |  |  | | | 8.33 | |  | |  | | | |
| ***Latin America*** | | | | | | | | | | | | | | | | | | | | | | | | | | | | | | | | | | | | | | | | | | | | | |  |
| Leiros GJ | | 2005 | | Argentina | | Biopsy, fixed tissue | | PCR | | 6,11,16,18 | Prostate hyperplasia | | 41  (41.46) | 30  (0.00) | 0 | | | 4.88 | 12.2 | | | 0 | |  | | |  | | |  | | |  | |  |  | | |  | |  | |  | | | |
| Martinez-Fierro ML | 2010 | | | Mexico | | Biopsy, fresh tissue | | nested PCR | | 33,45,52,58,66,68,83;44,81,cp6108 | No pathological evidence of PC | | 55  (20.00) | 75  (5.33) |  | | |  |  | | |  | |  | | |  | | |  | | |  | |  |  | | |  | |  | |  | | | |
| Silvestre RV | | 2009 | | Northern Brazil | |  | | PCR | | multiple | BPH | | 65  (3.08) | 6  (0.00) | 0 | | | 0 | 3.08 | | | 0 | | 0 | | | 0 | | | 0 | | | 0 | | 0 |  | | | 0 | | 0 | | 0 | | | |
| ***Mixed*** | | | | | | | | | | | | | | | | | | | | | | | | | | | | | | | | | | | | | | | | | | | | | |  |
| Strickler HD | | 1998 | | African-American,Italian | | Blood | | ELISAs | | 11,16 | BPH | | 63  (1.59) | 144  (4.86) |  | | | 0 | 1.59 | | |  | |  | | |  | | |  | | |  | |  |  | | |  | | |  | |  | | |

a. 5 studies only presented data of individual HPV types.

**Supplementary figure 1. Flow-chart for Studies Selection**

Potentially relevant original studies identified and screened for retrieval. (n=173)

Studies were excluded if no valid data on HPV infection in human prostate cancer were presented. (n=122)

If data subsets were published in more than one article, the previous one was excluded. (n=2)

Original studies retrieved for more detailed evaluation. (n=51)

Original studies retrieved for more detailed evaluation. (n=49)

Case reports, reviews and publications not in English were excluded. (n=3)

Studies with useful information were included for pooling the HPV prevalence in prostate cancer. (n=46)

The study was withdrawn for estimation of association between HPV infection and prostate cancer for lack of controls. (n=8)

Studies with useful information on both cases and controls were selected. (n=38)

The study was excluded automatically for ORs estimation of HPV infection on prostate cancer cases because of the absence of HPV detection in both cases and controls. (n=4)

Studies with useful information on both cases and controls were selected for ORs estimation of HPV infection on prostate cancer. (n=34)
